# Targeting abundant survivin expression in liposarcoma: subtype dependent therapy responses to YM155 treatment

**DOI:** 10.1007/s00432-021-03871-5

**Published:** 2021-12-03

**Authors:** Christian Vay, Philipp M. Schlünder, Levent Dizdar, Irene Esposito, Markus P. H. Ghadimi, Wolfram T. Knoefel, Andreas Krieg

**Affiliations:** 1 Department of Surgery (A), Heinrich-Heine-University and University Hospital Duesseldorf, Moorenstr. 5, Bldg. 12.46, 40225 Duesseldorf, Germany; 2 Institute of Pathology, Heinrich-Heine-University and University Hospital Duesseldorf, Moorenstr. 5, 40225 Duesseldorf, Germany; 3 Department of General, Visceral, Tumour, and Transplant Surgery, University of Cologne, Kerpener Strasse 62, 50931 Cologne, Germany

**Keywords:** Liposarcoma, Survivin, Inhibitor of apoptosis protein, Targeted therapy, Apoptosis

## Abstract

**Purpose:**

Liposarcoma (LPS) represent the largest group of malignant soft tissue tumours comprising a heterogeneous group of subtypes in which the degrees of chemoresistance and radiosensitivity strongly vary. Consequently, it is of utmost interest to establish novel therapeutic regimens based on molecular targets.

**Methods:**

Immunohistochemical staining of survivin was performed in tissue microarrays comprising 49 primary LPS specimens. LPS cell lines were treated with survivin antagonist YM155 and doxorubicin or etoposide alone as well as in combination. Changes in cell viability were investigated and the synergistic effect of a combined therapy analysed.

**Results:**

Immunohistochemistry revealed an abundant expression of survivin in LPS that significantly concurred with less-differentiated tumour subtypes and grading. In vitro, we demonstrated the impact of the survivin inhibitor YM155 on dedifferentiated LPS (DDLPS) and, even more imposing, pleomorphic LPS (PLS) tumour cell viability with a strong induction of apoptosis. A combined treatment of doxorubicin or etoposide with YM155 augmented the cytotoxic effects on DDLPS and PLS cells.

**Conclusion:**

These findings support the significant role of survivin in the oncogenesis and progression of LPS subtypes providing a rationale to target survivin in eligible in-vivo models and to pioneer clinical applications of survivin-specific substances unfolding their therapeutic potential in LPS patients prospectively.

**Supplementary Information:**

The online version contains supplementary material available at 10.1007/s00432-021-03871-5.

## Introduction

Liposarcoma (LPS) is among the most frequent types of soft tissue sarcoma with 50% of retroperitoneal localisation and 25% peripheral distribution (Crago and Brennan 2015).

Complete surgical resection is the central therapeutic approach towards all four main groups of LPS—well differentiated (WDLPS) and dedifferentiated liposarcomas (DDLPS) accounting for 60% of LPS cases, myxoid or round cell liposarcoma (MLPS) amounting to 20–30% (de Graaff et al. 2017), and pleomorphic liposarcoma (PLS) adding 5% to the total of LPS occurrences (Crago and Dickson 2016).

Sharing their fate of significant recurrence levels and a perspective of limited patient survival after surgery in a serious proportion of LPS patients, the various subtypes differ with regard to their biological attributes (Dalal et al. 2006; Lee et al. 2018). WDLPS with recurrence-free 5-year survival rates ranging from 93 to 100% (Kooby et al. 2004) often harbour 12q13-15 amplifications incorporating the MDM2 and CDK4 oncogenes. With more complex genomic aberrations frequently altering chromosomes 3, 11, and 19 (Crago et al. 2012), DDLPS stands out with a disease-specific 5-year-survival of 44%. MLPS are associated with a FUS-DDIT3 fusion protein acting as an aberrant transcription factor after frequent t(12;16) translocation. As 30–50% of PLS patients develop local tumour relapse and up to 50% of the cases prone for metastasis frequently evince losses of *TP53* and *Rb*, these frequent genetic aberrations remain difficult to be utilised therapeutically.

While surgery remains the primary therapeutic approach in all LPS entities, adjuvant treatment approaches strongly vary among the four subtypes due to significant differences in their susceptibility to adjuvant or neoadjuvant treatment regimens (Crago and Dickson 2016). Though their primaries are initially receptive for radio- and chemotherapy, even MLPS may recur with local relapse or systemic spread in up to 40% of the cases as reflected in considerably reduced survival rates (de Graaff et al. 2017). Whereas PLS appear partly chemosensitive, clinically DDLPS are frequently resistant to chemo- and radiotherapies or show only minor prognostic benefits (Italiano et al. 2012). Accordingly, the outcome of recurrent DDLPS remains extremely poor (Anaya et al. 2009; Park et al. 2009).

Despite the proven antiproliferative and cytotoxic properties of anthracyclines, alkylating agents, and topoisomerase inhibitors in several subtypes of LPS, undesirable side effects frequently occur and, as in other entities, tumour cell resistance develops during malignant progression providing only minor improvements of patient survival (Lee et al. 2017). For this reason, conventional systemic therapy components are sought to be potentiated by specifically targeting molecular pathways involved in cell proliferation and cell survival (Lee et al. 2018). While genomic alterations have been extensively investigated in soft tissue sarcoma and several substances have demonstrated their principal effectiveness in vitro (Barretina et al. 2010; Crago et al. 2016), none of these agents has been implemented in LPS treatment schemes so far.

As a substantial player in mitosis and programmed cell death, survivin is a promising candidate to contribute to the advancement of systemic LPS therapy and has been extensively reviewed recently by Wheatly and Altieri (Wheatley and Altieri^ 2019^). Among the five members of the inhibitor of apoptosis (IAP) family of multifunctional proteins, survivin is a highly conserved eukaryotic protein exerting its anti-apoptotic and mitotic activities in cytoplasm, mitochondria, and nuclei. Binding to the aurora B kinase, survivin contributes to the formation of the chromosomal passenger complex safeguarding the segregation of paired chromatid during mitosis. When XIAP is intercepted by survivin in the cytoplasm, caspase-9 inhibition blocks the activation of the apoptotic pathway. Simultaneously, survivin stimulates cell motility by the upregulation of α5-integrins (Wheatley and Altieri^ 2019^).

Physiologically expressed by proliferating cells during embryonal development and active in non-neoplastic adult thymic and placenta tissue only, survivin meets a central criterion for targeted tumour therapies as it is upregulated and overexpressed by most malignancies (Kanwar et al. 2013). Therefore, it has been considered as an oncotherapeutic target since its first description in 1997 (Ambrosini et al. 1997; Peery et al. 2017). As the expression of survivin has demonstrated its oncogenic and metastasis-enhancing potential, and, consequently, its prognostic relevance in several solid epithelial and endocrine malignancies (Mahotka et al. 2002; Span et al. 2004; Byun et al. 2007; McKenzie and Grossman 2012; Krieg et al. 2013a, 2013b; Werner et al. 2016, 2017; Brany et al. 2017; Dizdar et al. 2017a, 2017b, 2018), it has also been established as a relevant player in sarcoma formation and progression (Kappler et al. 2003; Ghadimi et al. 2011, 2012; Hingorani et al. 2013; Lusby et al. 2013; de Graaff et al. 2017). First hints that survivin might play a role in the tumour biology of LPS have been proposed by a study demonstrating abundant expression of survivin in PLS and MLPS specimens (Ghadimi et al. 2011; de Graaff et al. 2017). However, the biological and prognostic role of survivin still remains to be elucidated in all subtypes of LPS. Thus, the aim of our study was to shed light on the relevance of survivin as a biomarker according to the “*RE*porting Recommendations for Tumour *MARK*er Prognostic Studies (*REMARK*)” and to further evaluate its role as therapeutic target in LPS.

## Material and methods

### Patients

The study was approved by the local ethics committee at the Medical Faculty of the Heinrich-Heine-University Duesseldorf, Germany (institutional board review no. 3821), and carried out in accordance with good clinical practice and the Declaration of Helsinki (World Medical Association 2013). Each of the patients underwent tumour resection for primary LPS at the University Hospital Duesseldorf, Germany, in between 2001 and 2014. The tumours were staged and graded by pathologists according to the 8th edition of the TNM-classification recommended by the International Union Against Cancer (UICC) and World Health Organization (WHO). Tumours in which pathological staging was based on older TNM editions where re-classified according to the 8th edition. Follow-up data was collected until all experiments had been completed and overall survival was determined as the period from the date of surgery until the date of the last follow up or death of any cause. Patients with stage I-IV disease independently of the tumour localisation, neoadjuvant therapy and microscopic resection margin status who received surgery with curative intend were included in this study. Patients who had received only palliative chemotherapy after histological confirmation of LPS, who had deceased perioperatively within 30 days after surgery, or who had been lost to follow up were excluded from the study. Clinicopathological details were collected retrospectively from original pathological reports and patient case files. Follow-up data were retrieved before reviewing the experimental results.

### Tissue microarray and immunohistochemistry

Five tissue microarrays (TMA) were constructed from formalin-fixed and paraffin-embedded specimens comprising 49 samples of primary liposarcoma complemented by 15 lipomas, 13 samples from normal fatty tissue, and samples from other organs serving as positive controls (Packeisen et al. 2003), all of which originated from the Institute of Pathology, University Hospital Duesseldorf, Germany, where all cases had been reviewed by board-certified pathologists. A TMA contained two cylindrical specimens for each tumour sample from a donor block, five extracts from lipoma samples, five cylinders from normal fatty tissue, and three specimens deriving from other tissue types.

After preparing tissue sections with a thickness of 4 µm from the TMA, immunohistochemical staining was performed using the ZytoChem Plus HRP-DAB Kit (Zytomed Systems, Berlin, Germany) as described previously (Werner et al. 2016). In brief, after deparaffinisation and rehydration, epitope demasking was carried out at 95°C for 30ʹ using a 3% trisodium citrate dihydrate buffer equilibrated at pH 6.0, followed by cooling for 20’ to room temperature. Incubation of the tissue sections in 3% H_2_O_2_ phosphate-buffered saline (PBS, pH 7.4) for 10’ blocked endogenous peroxidase before the slides were rinsed three times for 2’ in PBS with 0.1% Tween-20 (Sigma-Aldrich, St. Louis, MO, USA). After blocking reagent was added to the sections for 10’ to block unspecific binding sites minimising background staining, the slides were washed in PBS with 0.1% Tween-20. Incubation with the rabbit primary polyclonal anti-survivin antibody (NB500-201; 1:750 dilution; Novus, Littleton, CO, USA), took 60’ at room temperature. Isotype controls with rabbit immunoglobulin fraction (Code X0903; 1:1,000 dilution; Dako, Glostrup, Denmark) served as negative controls. After triple rinsing the slides in PBS with 0.1% Tween-20, the sections were incubated with biotinylated secondary antibody and streptavidin-HRP conjugate, before 3,30-diaminobenzidine high contrast was added for 10’ in darkness resulting in epitope visualisation. Finally, tissue sections were counterstained with Mayer’s haematoxylin. Human colonic and tonsillar tissue specimens had been positively pretested for survivin expression served as positive controls.

Survivin staining intensities and percentage of chromogen positive cells were scored by two independent investigators according to the immunoreactivity score (IRS) reported by Remmele and Stegner (1987) without knowledge of histopathological parameters or patient survival outcome. Both investigators were experienced in the visual assessment and evaluation of the IRS. Differing ratings resulted in a re-examination of the respective samples by both investigators until a consensual scoring was reached.

### Cell culture

While the cell line Lipo-DUE1 was cultivated in RPMI 1640 Medium GlutaMax™ as previously described (Mersch et al. 2016), DDLPS cell line Lipo246A and PLS cell line PLS-1 kindly provided by Dina Lev (MTA No. MT2012-10,265) were maintained in DMEM 1× GlutaMax™ (both obtained from Gibco Life Technologies, Carlsbad, CA, USA). Both media were supplemented with 10% heat inactivated bovine FCS (Gibco Life Technologies, Carlsbad, CA, USA), penicillin, and streptomycin (both obtained from Biochrom GmbH, Berlin, Germany) to be kept in an atmosphere with 5% CO_2_ at 37°C. Cells were passaged routinely within seven days at a confluence of 80% by trypsinisation with 0.05% Trypsin/EDTA (Gibco Life Technologies, Carlsbad, CA, USA) and after washing with PBS (Gibco Life Technologies, Carlsbad, CA, USA).

### RNA quantification by real-time PCR

Total RNA was extracted from PBS washed LPS cells by RNeasy Mini Kits (Qiagen GmbH, Hilden, Germany) and concentrations were determined with the Infinite^®^ M200 microplate reader (Tecan Group Ltd., Mannedorf, Switzerland). After 5 µg of total RNA per sample were incubated with 0.5 µg Oligo(dT)18 Primer (Thermo Fisher Scientific, Waltham, MA, USA) for 10ʹ at 65 °C, cDNA was synthesized in 7 µl from a master mix of 0.5 µl Transcriptor Reverse Transcriptase, 4 µl Transcriptor RT Reaction Buffer 5× concentrated, 5 µl Protector RNase Inhibitor, and 2 µl dNTP Mix (Roche Diagnostics GmbH, Mannheim, Germany) for 30ʹ at 55 °C before the reverse transcriptase was inactivated for 5ʹ at 85 °C. After adjusting the cDNA concentrations to 2.5 ng/µl, triplicates from 2.5 µl of cDNA were mixed each with 12.5 µl FastStart TaqMan^®^ Probe Master (Roche Diagnostics GmbH, Mannheim, Germany), 0.25 µl probe solution (probe 11 and probe 60) from the Human Universal Probe Library Set (Roche Diagnostics GmbH, Mannheim, Germany) and 0.25 µl of forward (survivin: 5ʹ GCC CAG TGT TTC TTC TGC TT 3ʹ; GAPDH: 5ʹ GCC CAG TGT TTC TTC TGC TT 3ʹ) and reverse primers (survivin: 5ʹ AAC CGG ACG AAT GCT TTT TA 3ʹ; GAPDH: 5ʹ GCC CAA TAC GAC CAA ATC C 3ʹ) for quantitative real-time-PCR (survivin primers: Eurofins Scientific, Luxemburg, Luxemburg; GAPDH primers: Roche Diagnostics GmbH, Mannheim, Germany). GAPDH was used as internal reference gene. qPCR runs were conducted with the Chromo4 detector on a Dyad Disciple thermal cycler (Bio-Rad Laboratories Inc., Hercules, CA, USA) with 95 °C for 10ʹ, followed by 40 cycles of denaturation at 95 °C for 15ʹʹ and annealing and extension at 60 °C for 1ʹ. RNA expression values were calculated in relation to GAPDH and qPCR Human Reference Total RNA (Stratagene, La Jolla, CA, USA) in accordance with the 2^−ΔΔCT^-method as published by Livak and Schmittgen (2001).

### Flow cytometry

LPS cells were prepared for FACS analyses using the Molecular Probes FITC Annexin V/Dead Cell Apoptosis Kit (Thermo Fisher Scientific, Waltham, MA, USA). After treatment with YM155 (Selleck Chemicals LLC, Houston, TX, USA) in three different concentrations (30 nM; 100 nM; 300 nM) for 48 h, 1 × 10^6^ cells were washed in PBS and transferred to FACS vials (BD Biosciences, San Jose, CA, USA) before passing through the flow cytometry process in the BD FACSCanto™ device (BD Biosciences, San Jose, CA, USA) according to the manufacturer’s protocol. Cells were gated depending on the detected FITC-annexin and propidium iodide intensities, among which the double-positive cells were attributed to the apoptotic cell faction.

### Functional in vitro assays

MTS assays for cell viability were analysed in 96‑well culture plates with 1 × 10^4^ LPS cells seeded per well. After 24 h of cultivation as described above, cells were treated with various compound concentrations (30 nM; 100 nM; 300 nM) of YM155 (Selleck Chemicals LLC, Houston, TX, USA), doxorubicin (AppliChem GmbH, Darmstadt, Germany), etoposide (Merck KGaA, Darmstadt, Germany), or dimethyl sulfoxide (DMSO; Gibco Life Technologies, Carlsbad, CA, USA) in a minimum of three wells for 96 h, respectively. The CellTiter 96^®^ AQueous Non‑Radioactive Cell Proliferation Assay (Promega Corporation, Madison, WI, USA) was used to measure cell viability. All experiments were performed in triplicates and the mean IC_50_ was obtained based on the results of three independent experiments. For combinated treatment assays, the fractional products (FP) were determined as described by Webb (Webb 1963) with FP values < 1 representing synergistic effects, values = 1 additive effects, and values > 1 antagonistic effects.

Cell proliferation was determined by BrdU incorporation using a cell proliferation ELISA BrdU assay (Roche Diagnostics GmbH, Mannheim, Germany). Both assays were conducted according to the manufacturers’ protocols. Absorbances were measured with the Infinite^®^ M200 microplate reader (Tecan Group Ltd., Mannedorf, Switzerland), whereby absorbance values of YM155 treated cells were recorded as proportional to the absorbance of the corresponding DMSO treated control cells.

### Western blot analysis

1 × 10^5^ cultivated cells were harvested, washed in PBS, and transferred to 25 cm^2^ cell culture flasks for 24 h before being treated with YM155 (Selleck Chemicals LLC, Houston, TX, USA) or DMSO (Gibco Life Technologies, Carlsbad, CA, USA) for another 24 h. Then, cells were lysed in RIPA lysis buffer (Merck KGaA, Darmstadt, Germany) and incubated with protease inhibitor mix (cOmplete, Roche Diagnostics, Basel, Switzerland). Protein lysates were separated on SDS‑PAGE gels and blotted to nitrocellulose membranes (Thermo Fisher, Waltham, MA, USA) which were blocked with TBS‑T buffer containing 5% soluble nonfat dry milk (Nestlé, Vevey, Switzerland). After incubation with anti-survivin primary antibody (NB500-201; 1:1,000 dilution; Novus, Littleton, CO, USA) for 16 h at 4 °C and rinsing in TBS-T buffer, anti-rabbit IgG secondary antibody (HRP-linked Antibody #7074; 1:1,000 dilution; Cell Signalling Technology, London, UK) was added and incubated with 1.3 µl of Precision Protein™ StrepTactin-HRP (Bio-Rad Laboratories, Inc., Hercules, CA, USA) for 1 h. GAPDH as a loading control was detected by primary mouse anti-GAPDH antibody (Clone 6C5; 1:5,000 dilution; Abcam, Cambridge, UK) and marked by goat anti-mouse IgG (H&L (HRP); 1:5,000 dilution; Abcam, Cambridge, UK). Membranes were washed again in TBS-T buffer and developed with the Clarity Max™ Western ECL Substrate (Bio-Rad Laboratories, Inc., Hercules, CA, USA) and visualised with the VersaDoc Imaging System (Bio-Rad Laboratories GmbH, Munich, Germany). One representative western blot was selected for presentation after experiments were repeated thrice.

### Statistical analysis

Survivin protein expression in immunohistochemical stained TMAs was assessed by immunoreactivity scores (IRS) according to Remmele and Stegner (1987) and categorized into high (IRS ≥ 3) or low (IRS < 3) levels of expression according to the median IRS for survivin expression in all investigated LPS tissue samples. Correlations between non-parametrical data sets were analysed using the paired t-test, Mann‑Whitney-U test, Kruskal-Wallis test, or Dunn-Bonferroni test as indicated.The Fisher’s exact test, Cramér’s V, or, whenever appropriate, the Chi-square test were applied for categorical data. The correlation of numerical data with clinicopathological variables were examined applying the Mann‑Whitney-U test. Kaplan‑Meier curves were compiled and analysed using the log‑rank test (Mantel-Cox). Variables with a *p* value < 0.05 by univariate analysis were included in a multivariate Cox regression model using a backward selection. Computed analyses were conducted employing GraphPad Prism for Windows (version 5; GraphPad Software Inc., La Jolla, CA, USA), Microsoft Excel (version 14; Microsoft Corp., Redmond, WA, USA) and SPSS statistics for Windows (version 17.0; SPSS Inc., Chicago, IL, USA). A *p* < 0.05 was defined to indicate a statistically significant difference. Cramér’s *V* values of < 0.2 were interpreted as weak correlations, values ≥ 0.2–0.5 as moderate, and values > 0.5 as strong correlations.

## Results

### Primary liposarcoma—patients and outcome

According to our selection criteria, 49 samples from patients with primary LPS were included in the present study after undergoing surgical resection at our department between 2001 and 2014. The baseline characteristics of the included patients are summarized in Table 1. Patients’ median overall survival (OS) time was 41 months (range 4–146 months) resulting in a 5-year OS of 52.6%. At the end of follow-up 31 (63.3%) patients were still alive with a median follow-up time of 68 months (range 6–146 months).

**Table 1:** Patient characteristics (n = 49)

Variables	No. of patients (%)
Total	49
Age	
Median (range); years	58 (29–93)
Gender	
Male	29 (59.2)
Female	20 (40.8)
Localisation	
Head	1 (2)
Extremeties	21 (42.9)
Thorax	4 (8.2)
Abdomen	8 (16.3)
Retroperitoneum	15 (30.6)
Subtype	
WDLPS	18 (36.7)
MLPS	17 (34.7)
PLS	5 (10.2)
DDLPS	9 (18.4)
Grade	
G1	23 (46.9)
G2	10 (20.4)
G3	13 (26.5)
Undefined	3 (6.1)
Tumour stage	
T1	8 (16.3)
T2	18 (36.7)
T3	8 (16.3)
T4	15 (30.6)
Tumour size	
Median (range); cm	10 (2–33)
Neoadjuvant therapy	
Yes (EIA)	2 (4.1)
No	32 (65.3)
Unknown	15 (30.6)

Assessing the clinicopathologic parameters statistically, LPS affecting the deep soft tissue in the abdomen and retroperitoneum, which was to be distinguished from LPS localised within the superficial soft tissue of the head, extremities, or thorax, correlated with LPS subtypes PLS/DDLPS (*p* = 0.037; Cramér’s *V* = 0.417), higher tumour grade (*p* = 0.037; Cramér’s *V* = 0.417) and larger primary tumour size (T3/4) (*p* = 0.016; Cramér’s *V* = 0.344).

### Primary liposarcoma—survivin expression

**Fig. 1:**
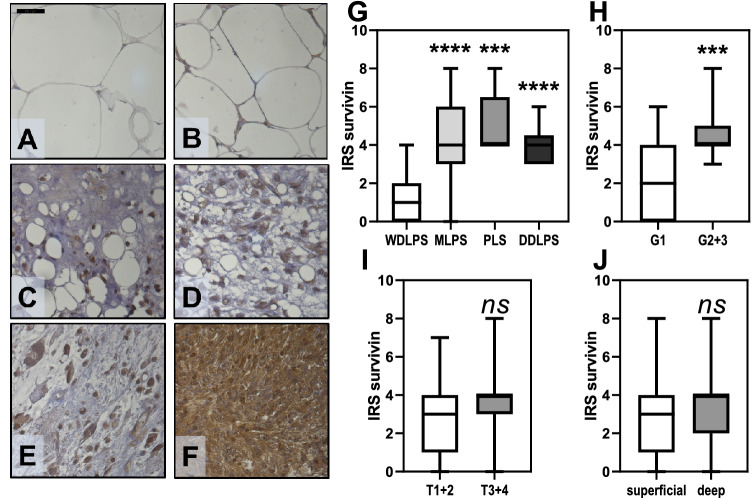
Expression of survivin in liposarcoma (LPS). Representative images after immunohistochemistry for survivin expression (DAB counterstained with Mayer’s haematoxylin) on tissue-microarrays (TMA) from **A** fatty tissue, **B** lipoma, **C** WDLPS, **D** MLPS, **E** PLS, and **F** DDLPS. Images were captured at 400fold magnification (scale bar indicates 25 μm). IRS distribution of survivin expression (statistical significances as indicated by asterisks): **G** While low (< 3) survivin expression was preponderant only in WDLPS, DDLPS, MLPS, and PLS shared significantly higher expression scores (≥ 3). **H** Low IRS values (< 3) correlated with G1-tumours, whereas less differentiated LPS (G2 + 3) exclusively classed with high IRS (≥ 3). **I, J** No significant differences in mean IRS values were apparent between grouped T-stages, or superficial versus deep localisation. DAB, 3,30-diaminobenzidine; TMA, tissue micro-array; WLDPS, well differentiated liposarcoma; MLPS, myxoid liposarcoma; PLS, pleomorphic liposarcoma; DDLPS, dedifferentiated liposarcoma; IRS, immunoreactivity score

After immunohistochemical staining, the IRS for survivin was assessed and the median IRS of 3 was defined as a cut-off to differentiate high from low expression. In contrast to the distinct LPS subtypes, survivin expression was undetectable in 13 fatty tissue samples and in 15 lipomas (Fig. 1A–F) [*p* < 0.001; Cramérs *V* = 0.655].

Whereas 83.3% of WDLPS samples demonstrated a low survivin expression, the remaining LPS subtypes (DDLPS 100%, MLPS 94.1%, PLS 100%) presented with exclusively or predominantly high expression (Table 2). Of note, high survivin expression levels also significantly correlated with higher grading (G2-3). In addition, when comparing the IRS as numeric variable across groups for each clinicopathological variable, we confirmed the association between survivin expression levels and LPS subtype or tumour grade (Fig. 1G–J).

**Table 2:** Correlation between survivin expression and clinicopathological markers in LPS

Variables	Low, *n* = 16 (%)	High, *n* = 33 (%)	*p* value
Age; years			
< 58	8 (50)	17 (51.5)	1.000^†^
≥ 58	8 (50)	16 (48.5)	
Gender			
Male	11 (68.8)	18 (54.5)	0.375^†^
Female	5 (31.2)	15 (45.5)	
Subtype			
WDLPS	15 (93.8)	3 (9.1)	< 0.001*
MLPS	1 (6.2)	16 (48.5)	
PLS	0 (0)	5 (15.1)	
DDLPS	0 (0)	9 (27.3)	
Tumour stage			
T1	4 (25)	4 (12.1)	0.437*
T2	7 (43.8)	11 (33.3)	
T3	2 (12.5)	6 (18.2)	
T4	3 (18.7)	12 (36.4)	
Grade			
G1	13 (81.3)	10 (30.3)	< 0.001*
G2	0 (0)	10 (30.3)	
G3	0 (0)	13 (39.4)	
Undefined	3 (18.7)	0 (0)	
Localisation			
Superficial	10 (62.5)	16 (48.5)	0.382^†^
Deep	6 (37.5)	17 (51.5)	

*WDLPS* well differentiated liposarcoma; *MLPS* myxoid liposarcoma, *PLS* pleomorph liposarcoma; *DDLPS* dedifferentiated liposarcoma; *low* *IRS* < mean; *high* *IRS* ≥ mean

^†^Fisher’s exact test

*Chi-square test

**Fig. 2:**
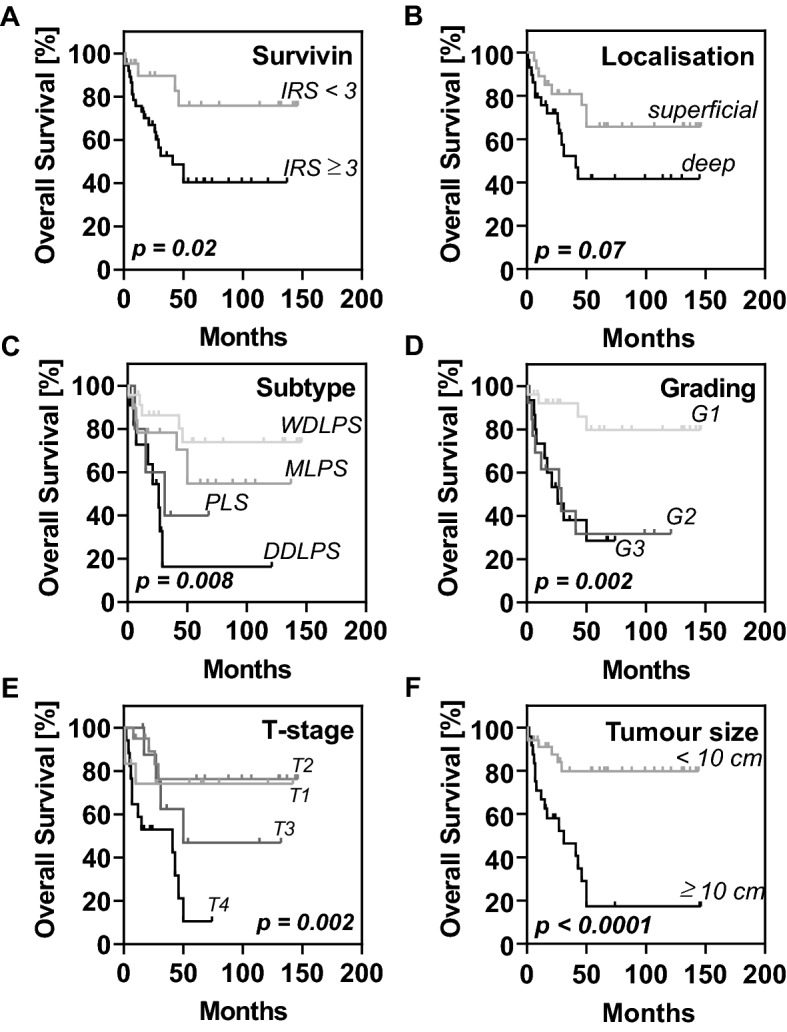
Overall survival of liposarcoma (LPS) patients according to survivin expression and clinicopathological variables. Kaplan–Meier survival curves illustrating the prognostic values of **A** survivin expression, **B** localisation, **C** LPS subtype, **D** tumour grading, **E** primary tumour depth (T-stage), and **F** tumour size. IRS, immunoreactivity score; WLDPS, well differentiated liposarcoma; MLPS, myxoid liposarcoma; PLS, pleomorphic liposarcoma; DDLPS, dedifferentiated liposarcoma

Next, we investigated whether survivin expression and clinicopathological variables were associated with patients’ overall survival. Thereby, we created Kaplan–Meier survival curves and performed survival analysis using log rank analysis as well as a Cox regression model (Fig. 2, Table 3).

**Table 3:** Overall survival analysis

Variables	HR	CI (lower–upper 95%)	*p* value
Univariate survival analysis			
Age at surgery	2.318	0.869–6.183	0.093
Sex	0.949	0.368–2.451	0.914
Subtype	1.960	1.289–2.980	**0.002**
T1/2 vs. T3/4	4.063	1.437–11.490	**0.008**
Grade (G1 vs. G2/3)	6.389	1.825–22.364	**0.004**
Localisation (superficial vs. deep)	1.805	0.707–4.607	0.217
Survivin expression	5.307	1.215–23.192	**0.027**
Multivariate survival analysis			
T1/2 vs. T3/4	4.391	1.479–13.036	**0.008**
Subtype	2.257	1.380–3.691	**0.001**

*CI* confidence interval; *HR* hazard ratio

*p* < 0.05 indicates statistical significance

Accordingly, univariate analysis revealed that high survivin expression (IRS ≥ 3) correlated with a shorter overall survival for patients with high expression levels of survivin in the tumour (HR 5.307, CI 1.215–23.192, *p* = 0.027) (Table 3). In addition, LPS subtype was identified as a prognostic parameter (HR 1.960, CI 1.289–2.980, *p* = 0.002). Moreover, high grade tumours (G2/3) (HR 6.389, CI 1.825–22.364, *p* = 0.004) and advanced T stages (T3-4) (HR 4.063, CI 1.437–11.490, *p* = 0.008) were significantly associated with patients’ prognosis.

Multivariate analysis finally identified the primary tumour depth (T stage) (HR 4.391, CI 1.479–13.036, *p* = 0.008) and tumour subtype (HR 2.257, 1.380–3.691, *p* = 0.001) as independent prognostic markers for the assessed cohort of primary LPS (Table 3).

### In-vitro effects of survivin in liposarcoma cells

To explore the biological role of survivin in LPS, we first analysed the base line expression in liposarcoma cell lines Lipo-DUE1 (DDLPS), Lipo246A (DDLPS), and.

**Fig. 3:**
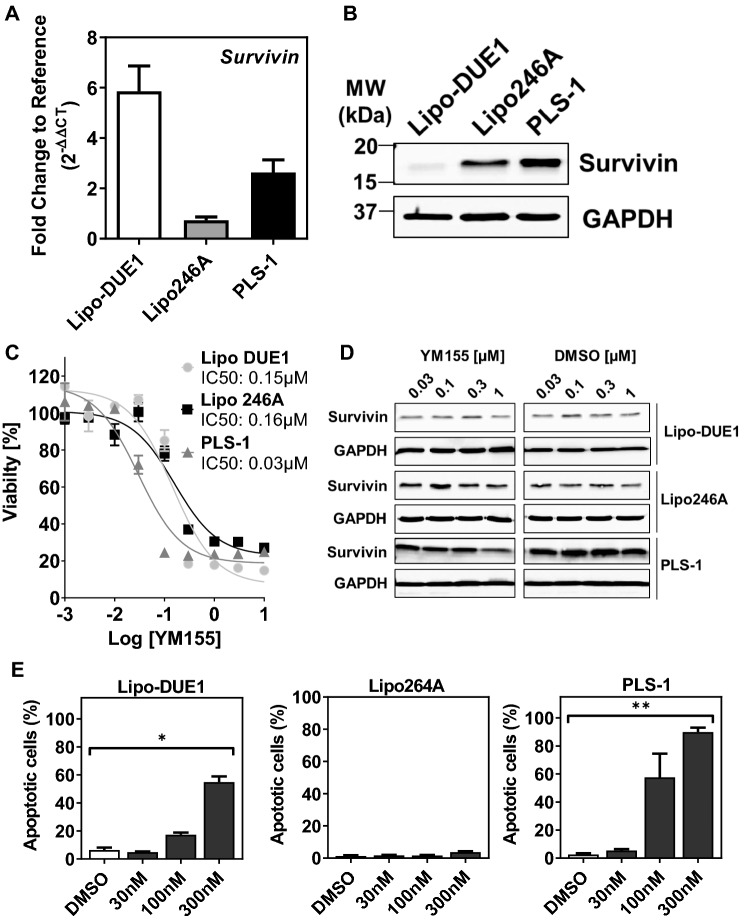
Antagonistic effects of sepantronium bromide (YM155) on survivin expression in LPS cell lines LipoDUE1, Lipo246A, and PLS-1. **A** Differing survivin RNA levels relative to GAPDH expression (2^−ΔΔCT^) as determined by qPCR. **B** Western blotting indicated variant base line protein expression of survivin in Lipo-DUE1, Lipo246A, and PLS-1. GAPDH expression served as a reference. **C** Dose-dependent cell viability decrease after 96 h of YM155 treatment in relation to DMSO controls with corresponding IC50 values as determined by MTS-assays. **D** Dose-dependent reduction of survivin protein expression by incubation with YM155 (1,000 nM) for 12 h was detectable only in PLS-1 cells as shown by western blotting compared to DMSO vehicle controls and in relation to GAPDH. **E** Percentage ranges of apoptotic cells after 48 h of YM155 treatment determined by FACS analyses: YM155 exerts significant apoptotic effects on Lipo-DUE1 (*p* < 0.05) and PLS-1 cells (*p* < 0.01) while Lipo246A cells remain largely unaffected. YM155, sepantronium bromide; LPS, liposarcoma

PLS-1 (PLS) by quantitative RT-PCR and western blot (Fig. 3A, B). While Lipo-DUE1 cells showed a significantly higher RNA expression with a mean 2^−ΔΔCT^ of 5.86 ± 2.47 (*p* = 0.0001; Kruskal–Wallis test), the protein expression in the cell line was comparatively weak. In contrast, PLS-1 as well as Lipo246A cells exhibited lower mean RNA expression levels of 0.73 ± 0.32 and an intermediate RNA-level amounting to 2.63 ± 1.22, respectively. At the same time, Lipo246A and more so PLS-1 protein levels exceeded the weak expression of survivin in Lipo-DUE1 cells.

To further elucidate the effect of a chemical inhibition of survivin, we incubated LPS cell lines with increasing concentrations of the small molecule antagonist YM155 for 96 h and measured cell viability by performing MTS assays (Fig. 3C). Incubation with YM155 resulted in a significant reduction of Lipo-DUE1, Lipo246A and PLS-1 cells in a dose-dependent manner with an IC50 of 0.15 µM, 0.16 µM, and 0.03 µM, respectively. Of note, a decrease in survivin protein levels became evident only in the more sensitive PLS-1 cells at 1 µM (Fig. 3D). To further assess the pro-apoptotic potency of survivin small molecule antagonist YM155, we again incubated LPS cell lines with increasing concentrations of YM155 and determined 24 h later the fraction of apoptotic cells by Annexin V/PI-staining and FACS (Fig. 3E). While Lipo-DUE1 cells showed an increase of apoptotic cells significantly correlating with the amount of administered YM155 reaching 17.3% at 100 nM and 54.8% at a 300 nM concentration (Kruskal-Wallis test, *p* = 0.0237; Dunn-Bonferroni test, *p* < 0.05), the treatment of Lipo246A cells with YM155 did not substantially affect the fraction of Annexin V and PI positive cells. In PLS-1 cells again, the increasing concentrations of YM155 were associated with higher proportions of apoptotic cells (Kruskal-Wallis test, *p* = 0.0156; Dunn-Bonferroni test, *p* < 0.01).

**Fig. 4:**
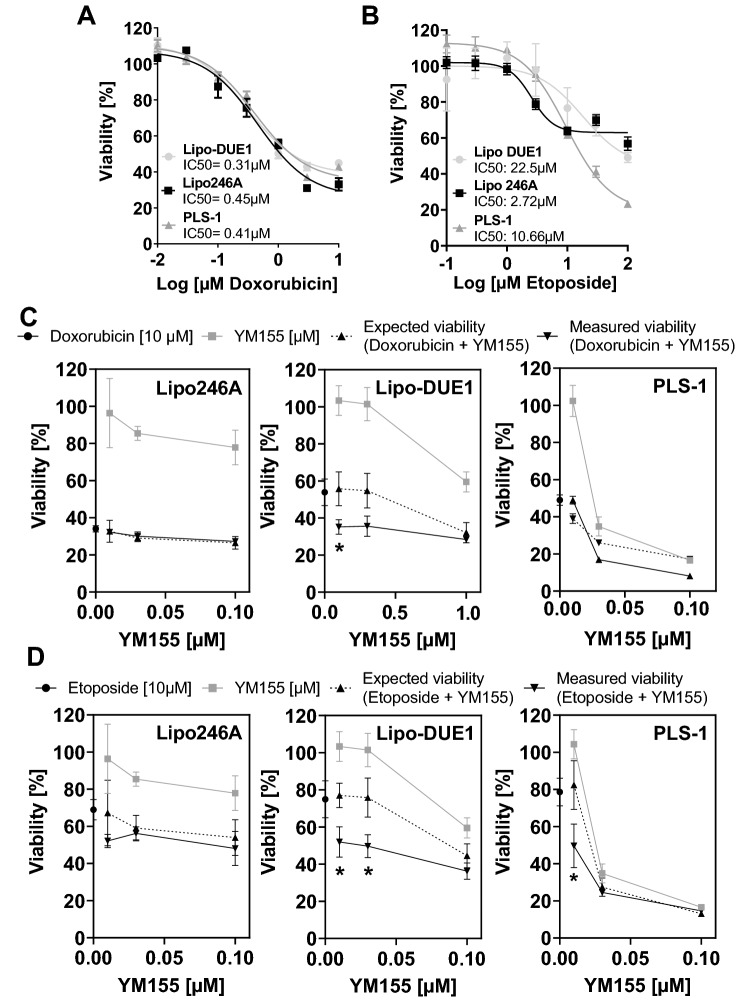
Synergistic effect of chemotherapeutic agents and survivin inhibitor YM155 in LPS cell lines. LPS cell viability by MTS assay after 96 h of treatment with **A** doxorubicin and **B** etoposide in various concentrations (0.01 µM; 0.03 µM; 0.1 µM; 0.3 µM; 1 µM; 3 µM; 10 µM) with IC50 values depicted: Increasing concentrations resulted in significant reductions of cell viability rates with uniform results for LipoDUE-1, Lipo246A, and PLS-1 cells during doxorubicin treatment. MTS assays for LPS cell lines treated with **C** doxorubicin 10 µM and **D** etoposide 10 µM in combination with YM155 in rising concentrations (0.01 µM; 0.03 µM; 0.1 µM) for 72 h. As indicated by asterisks, the fractional products (FP) between expected (cell viability calculated from simply adding up the effects of the single agents doxorubicin or etoposide and YM155) and measured viability rates of Lipo-DUE1 (DDLPS) cells indicate relevant sensitisation effects for combined treatment with YM155 and doxorubicin or etoposide (*indicates *p* < 0.05). YM155, sepantronium bromide; LPS, liposarcoma; DDLPS, dedifferentiated liposarcoma

Next, we tested the response of LPS cell lines to the clinically established cytotoxic single-agents doxorubicin and etoposide solitarily as well as in combination with YM155 (Fig. 4).

Both, doxorubicin and etoposide induced a significant decrease in cell viability of all investigated cell lines, whereby significantly higher concentrations were necessary for the latter (Fig. 4A, B). Of note, combinational treatment of doxorubicin (10 µM) with increasing concentrations of YM155 (10 nM; 30 nM; 100 nM) for 72 h demonstrated only in Lipo-DUE1 cells a synergistic effect for 10 nM (FP = 0.64) of YM155 when administered together with doxorubicin (Fig. 4C, Supplementary Table 1). However, in Lipo-DUE1 and PLS cells treated with 10 µM of etoposide, we observed a synergistic effect when combined with YM155 at nanomolar concentrations. (Fig. 4D, Supplementary Table 1).

## Discussion

Due to its yet not unequivocally understood but central function in cell cycle progression, apoptosis suppression, and cell migration, survivin has been established as a marker for chemoresistance in solid neoplasia showing promise as an inventive target for molecular therapy approaches (Wheatley and Altieri^ 2019^). While survivin expression proved to be significantly related to disease progression and patient outcome in several tumour entities, only a few studies have addressed its role in the formation and progression of LPS (LaPensee et al. 2007; Ghadimi et al. 2011; de Graaff et al. 2017).

In the present study, we investigated the characteristics of survivin expression in various liposarcoma subtypes collected from surgical specimens of 49 cases with primary LPS. For the cohort reflecting the general incidence, epidemiologic characteristics, and distribution of the respective LPS subtypes (Lee et al. 2018), the experimental results were correlated with the corresponding clinicopathological parameters including postoperative patient survival.

Residual tumour burden after resection, local recurrence, and metastasis have been described before as relevant prognostic markers in LPS as well as tumour localisation, subtype, grading, and size (Knebel et al. 2017), of which primary tumour depth and LPS subtype were identified as independent prognostic markers in the present study. In this context, DDLPS was predominantly found in deep abdominal compartments and, furthermore, high-grade tumours (G2-3) generally were larger in diameter than low-grade LPS (G1). Deep LPS localisation comprising predominantly retroperitoneal DDLPS was significantly related to a poor postoperative overall survival.

Survivin expression was determined by immunohistochemistry and evaluated according to Remmele and Stegner’s immunoreactive score taking into account both the intensity of staining and the percentage of positively stained cells (Remmele and Stegner 1987). Importantly, none of the samples taken from normal fatty tissue or lipoma expressed survivin. While WDLPS almost entirely shared low survivin expression scores, DDLPS, MLPS, and PLS consistently exhibited high levels of cytoplasmic as well as nuclear survivin closely corresponding with previously published series (Ghadimi et al. 2011; de Graaff et al. 2017). In addition, in our study the degree of survivin expression significantly reflected the tumour grading with 81.3% of the low-grade tumours (G1) showing low and 100% of high-grade tumours (G2-3) expressing high levels. Mean overall survival among patients with high level expression ranged below the average of the cohort reducing the 5-year-survival rates from 79% in patients with low survivin expression levels compared to 44% with high expression in their primary tumours.

While grading and survivin expression showed significance only in univariate analyses with respect to postoperative overall survival, the primary tumour depth as reflected by T stage and LPS subtype represented independent prognostic markers in the analysed cohort.

Beyond immunostaining for cellular survivin protein expression in primary liposarcoma subtypes, the expression of survivin was quantified in three LPS cell lines by qPCR on mRNA levels as well as by western blotting on protein levels: while the DDLPS cell line Lipo-DUE1 was characterized by high RNA-levels of survivin and low protein-levels—presumably due to post translational modifications—the two other cell lines Lipo246A (DDLPS) and PLS-1 (PLS) exhibiting only low to moderate survivin RNA levels impressed with substantially higher amounts of survivin protein expression.

Among the known survivin interacting agents, we chose the imidazolium compound sepantronium bromide (YM155), which supresses the survivin core promotor activity by disrupting the survivin binding ILF3/p54 complex and Sp1-DNA interaction required for survivin expression (Cheng et al. 2012; Yamauchi et al. 2012), as the most promising substance to assess its potential effectiveness in LPS. The usefulness of YM155 in soft tissue sarcoma had been described before for human malignant peripheral nerve sheath tumours, MLPS and osteosarcoma (Ghadimi et al. 2012; Zhang et al. 2016; de Graaff et al. 2017). Our experimental treatment of three LPS cell lines with YM155 effectively resulted in significant reductions of cell viability in DDLPS and PLS cell lines already by nanomolar concentrations, even though protein levels were only perceptibly decreased in PLS-1 cells. Analogously, effective suppression of tumour cell growth by YM155 has been described before for MLPS cells sharing strong nuclear aggregations of survivin as the administration of YM155 resulted in a 70–90% decrease of viable cells in two of three cell lines assessed (de Graaff et al. 2017). Apoptosis, however, had not been induced in MLPS by YM155.

Determining the fraction of Annexin V/PI positive cells after YM155 exposition revealed that in the cell lines Lipo-DUE1 and, particularly, in PLS-1 the percentage of apoptotic cells significantly rose to more than 55% and 90%, respectively. In contrast, YM155 treatment of Lipo246A cells did not enhance apoptosis in a considerable proportion of cells.

Treatment of the three cell lines with the LPS approved chemotherapeutic agents doxorubicin and etoposide (Tacar et al. 2013), respectively, resulted in a significant dose-dependent reduction of viable tumour cells up to 50–70%. This effect had been demonstrated similarly for the metastatic liposarcoma cell line SW872 (LaPensee et al. 2007) treated with doxorubicin resulting in a decrease of cell viability of 80–90% and MLPS cell lines (402,091; 1,765,092; DL-221) with a cell-death induction rate of more than 80% (de Graaff et al. 2017). Clinically, as far as 44% of MLPS patients may respond to doxorubicin (Patel et al. 1994), whereas PLS patients have not demonstrated prognostic improvements after single anthracycline treatment (Eilber et al. 2004). In line with the observation that YM155-mediated decrease of survivin activity has attenuated chemoresistance in other malignancies (Koike et al. 2014; Guo et al. 2015), in the present study combined treatment of doxorubicin and etoposide with YM155 demonstrated a drug-depending synergistic effect in the DDLPS Lipo-DUE1 as well as the pleomorphic PLS-1 cell lines at low concentrations of YM155. The impressive reduction of tumour cell viability and the anti-apoptotic effects of single agent YM155 in DDLPS and PLS cells as well as the potential function as a drug sensitizer challenged in our experiments highlights its potential of targeting survivin in LPS. Of note, the safety and therapeutic efficacy of dendritic cells expressing recombinant survivin are now under investigation in high-risk soft tissue sarcoma in a phase I/II clinical trial (NCT01898663). Another active phase I clinical trial targets survivin by cytotoxic T-lymphocytes in rhabdomyosarcoma (NCT02239861).

Notwithstanding the limitations of the study inter alia its retrospective design and the absence of in-vivo experiments, our findings underscore the potential role of survivin in the oncogenesis and progression of the distinct LPS subtypes providing a rationale to target survivin in appropriate LPS in-vivo models. Its almost exclusive presence with significant expression levels in tumour tissues maintains the special attractiveness of survivin for targeted therapy approaches in LPS as in other malignancies.

## Conclusion

In this study we demonstrated that survivin expression correlated with tumour subtype and grading in LPS. In addition, we showed the effect of survivin inhibitor YM155 on DDLPS and PLS viability. Importantly, a combination of doxorubicin or etoposide with YM155 synergistically enhanced the cytotoxic effects on DDLPS and PLS cells. Thus, our results further endorse the pre-clinical advancement of novel compounds and warrant clinical applications of promising survivin-specific substances to investigate their therapeutic efficacy in LPS patients prospectively.

## Supplementary Information

Below is the link to the electronic supplementary material.

Supplementary file1 (PDF 97 KB)

## Data Availability

The datasets used and/or analysed during the current study are available from the corresponding author on reasonable request.
