# Supplementary material for: Targeting abundant survivin expression in liposarcoma: subtype dependent therapy responses to YM155 treatment
Source: J Cancer Res Clin Oncol. 2021 Dec 3;148(3):633–45. doi: 10.1007/s00432-021-03871-5 (PMC8881260; doi:10.1007/s00432-021-03871-5)
Supplement: Supplementary file 1 — Supplementary file1 (PDF 97 KB) [file 432_2021_3871_MOESM1_ESM.pdf]

Targeting Abundant Survivin Expression in Liposarcoma – Subtype Dependent Therapy Responses to YM155 Treatment

Christian Vay, Philipp M. Schlünder, Levent Dizdar, Irene Esposito, Markus Philipp Hussein Ghadimi, Wolfram T. Knoefel, Andreas Krieg

Journal of Cancer Research and Clinical Oncology

Correspondence: Andreas Krieg, Department of Surgery (A), Heinrich-Heine-University and University Hospital Duesseldorf, Moorenstr. 5, Bldg. 12.46, 40225 Duesseldorf, Germany; Phone: + 49 211 81 19251; Fax: + 49 211 81 19205; Email: andreas.krieg@med.uni-duesseldorf.de; ORCID ID: https://orcid.org/0000-0003-2496-1079

Supplementary Table 1 Fractional Product (FP) calculations for combinations of YM155 and doxorubicin or etoposid

	**doxorubicin**	**etoposide**
**Lipo-DUE1**		
YM155 (100 nM)	0.91	0.83
YM155 (30 nM)	0.68	0.67
YM155 (10 nM)	0.64	0.68
**Lipo246A**		
YM155 (100 nM)	1.04	0.92
YM155 (30 nM)	1.03	0.96
YM155 (10 nM)	1.01	0.84
**Lipo246A**		
YM155 (100 nM)	2.12	1.13
YM155 (30 nM)	1.54	0.93
YM155 (10 nM)	0.79	0.61

The measured viability of a combinational treatment is related to an expected viability calculated from the viability of treatment with single drugs, using the fractional product method of Webb. Values < 1 indicate synergism, = 1 indicate additive effects and > 1 indicate antagonism
